# Music, emotion, and time perception: the influence of subjective emotional valence and arousal?

**DOI:** 10.3389/fpsyg.2013.00417

**Published:** 2013-07-17

**Authors:** Sylvie Droit-Volet, Danilo Ramos, José L. O. Bueno, Emmanuel Bigand

**Affiliations:** ^1^Laboratoire de Psychologie Sociale et Cognitive, University Blaise Pascal, CNRSClermont-Ferrand, France; ^2^Departamento de Mùsica, Federal University of ParanáParaná, Brazil; ^3^Faculdade de Filosofia, Ciências e Letras, University of São PauloSão Paulo, Brazil; ^4^Laboratoire d'étude de l'apprentissage et du développement, University of Burgundy, CNRSDijon, France

**Keywords:** time perception, music, emotion, valence, arousal

## Abstract

The present study used a temporal bisection task with short (<2 s) and long (>2 s) stimulus durations to investigate the effect on time estimation of several musical parameters associated with emotional changes in affective valence and arousal. In order to manipulate the positive and negative valence of music, Experiments 1 and 2 contrasted the effect of musical structure with pieces played normally and backwards, which were judged to be pleasant and unpleasant, respectively. This effect of valence was combined with a subjective arousal effect by changing the tempo of the musical pieces (fast vs. slow) (Experiment 1) or their instrumentation (orchestral vs. piano pieces). The musical pieces were indeed judged more arousing with a fast than with a slow tempo and with an orchestral than with a piano timbre. In Experiment 3, affective valence was also tested by contrasting the effect of tonal (pleasant) vs. atonal (unpleasant) versions of the same musical pieces. The results showed that the effect of tempo in music, associated with a subjective arousal effect, was the major factor that produced time distortions with time being judged longer for fast than for slow tempi. When the tempo was held constant, no significant effect of timbre on the time judgment was found although the orchestral music was judged to be more arousing than the piano music. Nevertheless, emotional valence did modulate the tempo effect on time perception, the pleasant music being judged shorter than the unpleasant music.

Music is a powerful emotional stimulus that changes our relationship with time. Time does indeed seem to fly when listening to pleasant music. Music is therefore used in waiting rooms to reduce the subjective duration of time spent waiting or in supermarkets to encourage people to stay for longer and buy more. A number of studies have indeed shown that a period of waiting is judged shorter when there is accompanying music than when there is none (e.g., Stratton, 1992; North and Hargreaves, 1999; Roper and Manela, 2000; Guegen and Jacob, 2002) and that this subjective shortening of time appears to be greater when the subjects enjoy this accompanying music (Yalch and Spangenberg, 1990; Lopez and Malhotra, 1991; Kellaris and Kent, 1994; Cameron et al., 2003). These findings raise the question: What are the musical parameters that produce emotions and change our time judgments?

Music is a complex structure of sounds whose different parameters can affect the perception of time. Much of the published literature considers that the major cause of subjective time distortions in response to music is due to the temporal regularities of musical events. According to Jones and Boltz (1989), the effect of music on time estimation is due to the perceptual expectancies that listeners develop when they hear a piece of music. The way musical accents are patterned through time leads listeners to anticipate the timing and nature of incoming events. They thus judge time to be shorter when these events occur earlier in the piece than expected, and longer when they occur later. This finding highlights the influence exerted by musical structures (pitch and rhythmic structure) on attention during the estimation of musical time (see also Tillmann et al., 2007; Firmino and Bueno, 2008; Firmino et al., 2009).

However, without rejecting the important role of musical structure, other researchers mention the critical role of the emotional qualities of music *per se*. Indeed, music is remarkable in its ability to induce emotions in listeners (Juslin and Sloboda, 2001). Many studies conducted over the last decade have indeed demonstrated the consistency of emotional responses to music (e.g., Peretz et al., 1998; Bigand et al., 2005). However, the musical structure of a piece of music may also induce emotions in listeners, with the result that musical structure and emotional qualities cannot be easily dissociated. Quite surprisingly, only a small number of studies in the fields of music cognition and time perception have investigated the influence of musical structure and emotional qualities. The present study therefore focuses on the potential influence of the emotional qualities of musical pieces on time judgment.

As far as the emotional qualities of musical pieces are concerned, the musical mode has been found to have robust effects on perceived emotion, with pieces perceived as sounding happy when played in a major key and sad when played in a minor key (e.g., Crowder, 1984; Peretz et al., 1998; Fritz et al., 2009). Influences of mode on time estimation have been reported in studies using stimulus durations of several minutes (Kellaris and Kent, 1992; Bisson et al., 2009). For instance, Bisson et al. (2009) showed that the duration of a joyful musical piece (taken from Bach's Brandenburg Concertos) was overestimated compared to that of a sad piece (Barber's Adagio for Strings). However, given that the two emotions were instantiated only by two entirely different pieces, it is difficult to be sure that this difference in time estimation was not caused by other structural parameters (rhythm, meter, tempo) that are not necessarily directly related to emotion. Indeed, a piece of music in a major key that is judged happy is often associated with a fast tempo, whereas pieces written in a minor key tend to be played in a slow tempo. In such cases, the critical factor may thus be the musical rhythm rather than the mode *per se*. Moreover, two recent studies conducted using shorter stimulus durations and various temporal paradigms failed to find any significant effect of major *vs*. minor mode on time estimation. Using a retrospective time estimation paradigm, in which the participants were informed that they had to estimate time only after the presentation of the event, Bueno and Ramos (2007) did not observe any differences in time estimation between a musical piece (64.3 s) played in major and minor mode. Similarly, using a prospective time estimation paradigm (i.e., a temporal bisection task) in which the subjects were instructed that they would have to estimate time, Droit-Volet et al. (2010a) did not report a significant effect of mode on time judgments when the musical excerpts were matched on all parameters except for mode. Consequently, these authors concluded that the emotional valence of music may have little influence on time perception, at least when all other parameters, such as pitch structure, are held constant.

Finally, we can assume that it is the structure of musical pieces, which is indirectly responsible for inducing emotions, that affects the perception of time rather than the emotional valence *per se*. Using simple sequences of clicks, numerous studies on timing have shown that faster rhythms lead to longer time estimates than slower rhythms (e.g., Treisman et al., 1990, 1992; Penton-Voak et al., 1996; Droit-Volet and Wearden, 2002; Ortega and López, 2008). To explain these results, the various authors argue that the sequence of clicks increases the level of arousal that makes the internal clock run faster. According to the internal clock models (Treisman, 1963; Gibbon, 1977; Gibbon et al., 1984), the raw material for the representation of time consists of pulses that are emitted by a pacemaker-like system and accumulated in a counter during the presentation of the stimulus duration. Consequently, when the internal clock speeds up under the influence of clicks, more pulses are accumulated for a given duration, and time is judged longer. It therefore seems reasonable to consider that the critical factor in time distortions with music is the musical tempo that also seems to affect the emotional arousal. As explained in Droit-Volet and Meck (2007), an increase in the arousal level with emotional stimuli is associated with a speeding up of the internal clock, with the result that time is judged longer. According to psychophysiological studies that have used standardized emotional material (e.g., Greenwald et al., 1989; Lang et al., 1999), the arousal dimension of emotional stimuli corresponds to a subjective state ranging from calm-relaxed to excited-stimulated. An increase in arousal level is indeed associated with physiological activation of the autonomic nervous system (Juslin and Västfjäll, 2008). In addition, it has been demonstrated that physiological measures of arousal (heart rate or skin conductance) are correlated with self-assessment of arousal on the Self-Assessment Manikin Scale (SAM, Lang, 1980; Lang et al., 1999). Therefore, one aim of the present study was to examine the effect of different musical pieces on time estimation by comparing the effects of different tempi. Tempo, however, is thought to play a role in the subjective emotional arousal assessed by the SAM scale (Lang, 1980) and not in affective valence.

In music, the concept of emotional valence may be understood in two different ways (Bigand et al., 2005). First, valence may be thought in terms of an opposition between “sad” and “happy” music, that is to say, between negative and positive emotions (see also Juslin and Västfjäll, 2008). One effective way of implementing this opposition is to contrast music in major and minor keys. However, neither Bueno and Ramos (2007) nor Droit-Volet et al. (2010a) found any effect of mode on the perception of time. Second, valence may be viewed in terms of “pleasant” and “unpleasant” music. In this perspective, music qualified as “sad” could easily be experienced as very pleasant (Droit-Volet et al., 2010a). In a study run by Blood et al. (1999), extremely pleasant music was found to stimulate the reward circuit of the brain. Consequently, sad music can also bring about this rewarding effect. It is therefore possible that the valence of musical stimuli contributes differently to time estimation depending on whether the implemented contrast is between negative/positive emotions or pleasant/unpleasant emotions. In the present study, we manipulated this aspect of musical valence (pleasant vs. unpleasant) by inverting the amplitude envelope of the musical pieces. More precisely, the structure of the musical stimuli was changed by playing the sound wave either normally or backward. We expected this backward version to render the music unpleasant for two reasons: it destroys the musical relationships between tones and it modifies the amplitude envelope of each musical tone.

In sum, in a first experiment, the participants performed a temporal bisection task composed of a training and a testing phase (Allan and Gibbon, 1991; Wearden, 1991; Droit-Volet and Wearden, 2001). In the training phase, the participants were initially trained to respond “short” or “long” for a short and a long standard duration presented in the form of a white noise. In the testing phase, they were then presented with different comparison stimulus durations, equal to the short or the long standard duration, or of intermediate value. Their task was to judge whether each comparison duration was more similar to the short or to the long standard duration. However, in the testing phase, the comparison stimulus durations were not a white noise, but musical pieces whose tempo (fast vs. slow) and valence (normal vs. backward) were both manipulated. Our main hypothesis was that the psychometric function in bisection (proportion of long responses plotted against comparison durations) would be shifted toward the left for the musical pieces with a fast tempo compared to that for the musical pieces with a slow tempo, the participants responding more often long for the former. Using emotional scales similar to those employed in the SAM scale developed by Lang et al. (1999), we also verified whether tempo was associated with the subjective emotional arousal and the normal vs. backward opposition with the subjective emotional valence.

## Experiment 1

### Method

#### Participants

Forty undergraduate students (27 women and 13 men, mean age = 19.2, *SD* = 1.02) at Burgundy University, France, participated in this experiment.

#### Material

The participants sat in a quiet laboratory room in front a PC computer that controlled the experimental events and recorded the responses via E-prime. The participant's responses consisted in pressing the “D” or the “K” keys of the computer keyboard. The participants also listened to the stimuli through headphones which were connected to the computer. The stimuli to be timed consisted of musical sequences. Each excerpt was recorded using Cubase 4 musical software (Steinberg). A set of 5 different musical piano pieces were used as the stimuli to be timed. The same 5 musical pieces, with identical musical parameters, were subjected to two types of manipulation: one for the tempo and the other for the valence. As far as tempo is concerned, we changed the tempo from slow (72 beats per min) to fast (184 beats per min). To manipulate the valence, we changed the structure of the stimuli by playing the sound wave either normally or backward. Manipulating both the tempo (slow vs. fast) and the valence (original vs. backward) for the 5 musical pieces resulted in the generation of 20 musical sequences for use in this experiment.

#### Procedure

The participants performed a temporal bisection task composed of two phases: training and test phase. In the training phase, the participants were presented with a short (*S*) and a long (*L*) standard duration presented in the form of a white noise. There were 16 trials, 8 for each standard duration, presented in a random order. In this phase, the participants were trained to respond “short” for *S* and “long” for *L*, by pressing the corresponding key. The button press order was counterbalanced across subjects. Only participants who obtained at least 70% correct responses were included in the testing phase. In this testing phase, the participants were presented with 7 comparison durations presented in the form of the musical pieces described above: one for each comparison duration similar to *S* or *L*, and one for the 5 intermediate comparison durations. For each musical piece, the participants must respond whether its comparison duration was more similar to *S* or to *L*. The test phase consisted of 280 trials presented in 2 blocks of 140 trials each: 10 trials for the musical stimuli (2 × 5 different musical pieces) with two types of tempo (slow vs. fast) and two types of valence (normal vs. backward) for each of the 7 comparison durations. The trials were presented randomly within each block. In addition, the participants were divided into two groups as a function of the duration range used: 0.5/1.7 or 2.0/6.8 s. For the shorter duration range, *S* was 0.5 s and *L* 1.7 s. The comparison durations were 0.5, 0.7, 0.9, 1.1, 1.3, 1.5, and 1.7 s. For the longer duration range, *S* and *L* were 2.0 and 6.8 s, and the comparison durations 2.0, 2.8, 3.6, 4.4, 5.6, 6, and 6.8 s. In each condition, the participants were instructed not to count the time (for the methods used to prevent counting, see Rattat and Droit-Volet, 2012).

After the bisection task, the participants were asked to evaluate the emotional qualities of the musical stimuli. More precisely, they heard each musical stimulus and rated its affective valence from “unpleasant” to “pleasant” and its arousal dimension from “calm” to “exciting” on a 9-point scale (range 1–9) similar to that used in the SAM by Lang et al. (1999). The two emotional scales were randomly presented. The presentation duration of each musical stimulus was at the mid-point between the two standard durations employed in the bisection task. In the 0.5/1.7 and the 2.0/6.8 s duration conditions, the participants thus gave their emotional judgments for stimuli of 1.1 and 4.4 s, respectively.

## Results and discussion

### Emotional evaluation of musical stimuli

Table 1 displays the emotional ratings for the music, presented for 1.1 and 4.4 s, as a function of the affective and arousal dimensions of each version of the pieces tested, when these were presented forward (original version) or backward and at a slow or fast tempo.

**Table 1:** **Mean and standard deviation of ratings of arousal and pleasantness (9-point scale) for musical excerpts presented in their original and backward version with a fast and a slow tempo for a 1.1 and a 4.4-s duration**.

**Music**	**Arousal**				**Pleasantness**			
	**1.1 s**		**4.4 s**		**1.1 s**		**4.4 s**	
	***M***	***SD***	***M***	***SD***	***M***	***SD***	***M***	***SD***
Original fast	7.31	1.83	8.22	0.81	7.60	1.24	7.98	1.05
Original slow	3.26	1.41	3.28	1.33	6.30	1.87	6.90	1.43
Backward fast	6.62	1.22	6.27	1.17	3.52	1.74	3.43	1.83
Backward slow	4.29	1.84	3.18	1.55	2.80	1.67	2.27	1.19

An ANOVA was run on each of the pleasantness and arousal ratings, with duration, backward version and tempo as within-subject factors. There was a significant main effect of both version, *F*_(1, 40)_ = 168.16, *p* < 0.05, η^2^ = 0.81, and tempo, *F*_(1, 40)_ = 60.99, *p* < 0.05, η^2^ = 0.60, on pleasantness. The main effect of duration, *F*_(1, 40)_ = 0.10, *p* > 0.05, was not significant, thus indicating that the presentation duration of the music (short or long) did not affect pleasantness. There was no significant interaction involving these different factors (all *p* > 0.05). In line with our hypothesis, our results thus showed that the normal version of the music was clearly judged to be more pleasant (7.20) than the backward version (3.01). The fast tempo was also judged more pleasant than the slow tempo (5.63 vs. 4.57), although the ratings tended more toward a median value on the 9-point scale.

As far as the arousal ratings are concerned, the ANOVA showed a significant main effect of tempo, *F*_(1, 40)_ = 234.50, *p* < 0.05, η^2^ = 0.85, thus demonstrating that the music played at a fast tempo was judged more arousing than the music played at a slow tempo (7.11 vs. 3.5). There was, however, a significant interaction between the tempo and the backward version, *F*_(1, 40)_ = 41.88, *p* < 0.05, η^2^ = 0.51. Tempo did not significantly interact with any other factor (all *p* > 0.05). This significant interaction indicated that, at the fast tempo, the participants judged the music to be more arousing in its normal than in its backward version (7.77 vs. 6.44, *F*^1^_(1, 41)_ = 18.22, *p* < 0.05, η^2^ = 0.31). In contrast, at the slow tempo, there was no difference between the normal and the backward version (3.27 vs. 3.73, *F*_(1, 41)_ = 1.83, *p* > 0.05). In addition, the ANOVA found a significant interaction between the backward version and the duration, *F*_(1, 40)_ = 4.31, *p* < 0.05, η^2^ = 0.10. The original music was judged more arousing than the backward music when the presentation duration was long (5.75 vs. 4.74, *F*_(1, 20)_ = 12.93, *p* < 0.05, η^2^ = 0.39), while both forms were judged to be similarly arousing when the duration was shorter (3.27 vs. 3.73, *F*_(1, 20)_ = 0.11, *p* > 0.05). However, the arousal rating did not exceed 5.75 on the 9-point scale. No other significant effect was found. In summary, in line with our hypotheses, the results suggested that the type of presentation (original vs. backward) was the main factor affecting the assessment of the valence of the musical pieces, and the tempo the main factor affecting the level of arousal induced by music, although with the fast tempo, the subjective arousal increased more with the normal than with the backward version of musical pieces.

### Temporal bisection

**Figure 1:**
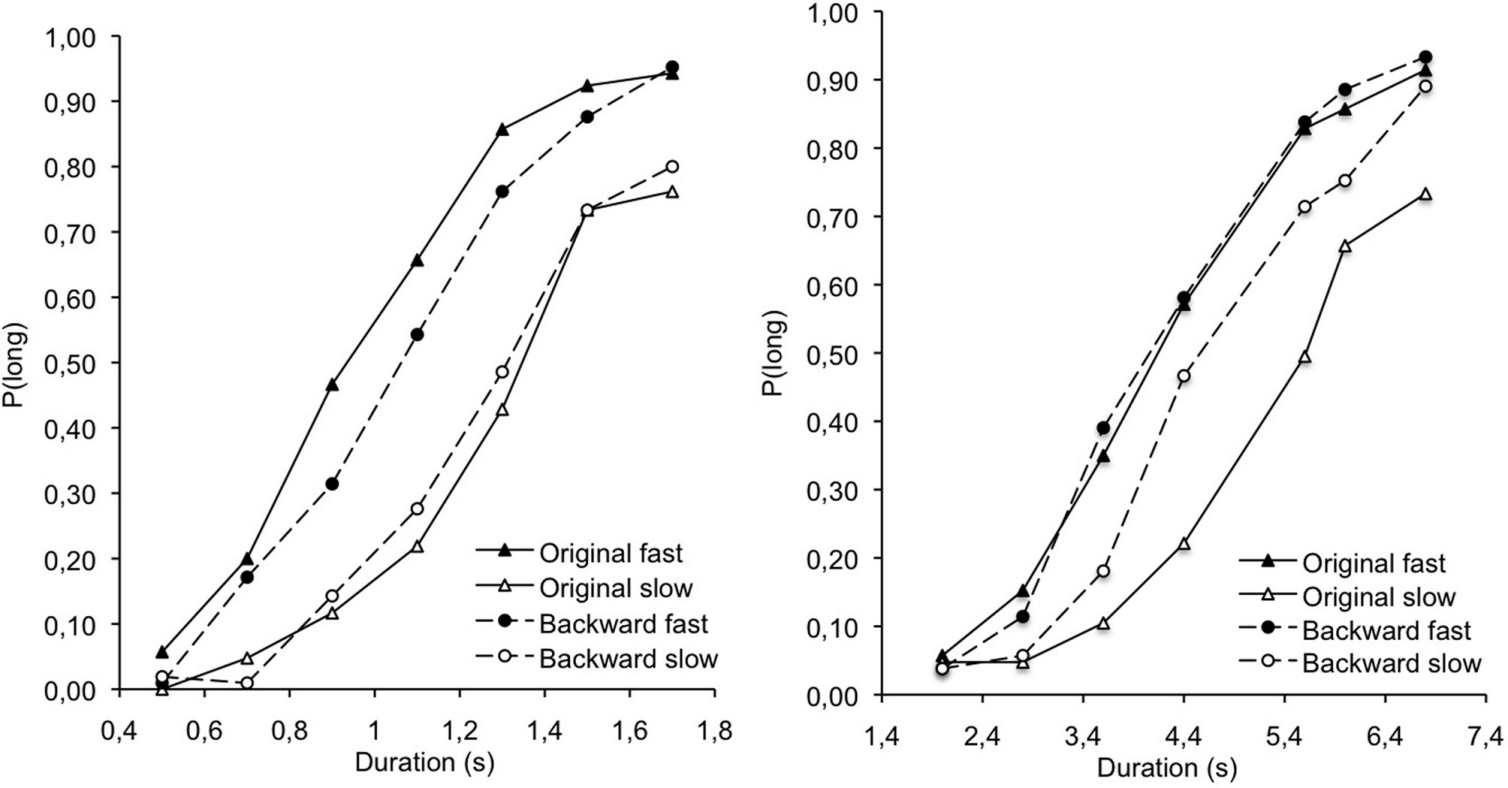
**Proportion of long responses plotted against stimulus duration for the original and the backward music with a slow and fast tempo in the 0.5–1.7 and the 2.0–6.8 s duration conditions**.

Figure 1 presents the proportion of long responses [*p*(long)] plotted against the comparison durations for the different types of musical pieces, which were judged to be high or low-arousing as a function of their tempo (fast vs. slow, respectively) and pleasant or unpleasant as a function of their version (original vs. backward). An examination of Figure 1 reveals that the major factor that produced time distortions was the tempo. Indeed, the musical stimuli were systematically judged longer with a fast than a slow tempo. To examine the bisection performance in more detail, we calculated two indexes: The point of subjective equality, also called the bisection point (BP), and the Weber Ratio (WR) (Table 2). The former is the stimulus duration (t) that gives rise to *p*(long) = 0.50. The WR is an index of time sensitivity. It is the Difference Limen (t[*p*(long) = 0.75] − t[*p*(long) = 0.25] /2) divided by the BP. The lower the WR value, the higher the sensitivity to time. The regression method originally used by Church and Deluty (1977) and subsequently employed by other authors (e.g., Wearden and Ferrara, 1996; Droit-Volet and Wearden, 2002) was used to calculate these 2 temporal indexes.

**Table 2:** **Means and standard deviation of the Bisection Points and Weber Ratios for musical excerpts presented in their original and backward version with a fast and a slow tempo in the 0.5/1.7 and the 2.0/6.8-s duration condition**.

**Music**	**Bisection point**		**Weber ratio**	
	***M***	***SD***	***M***	***SD***
**0.5/1.7 S**				
Original fast	0.87	0.18	0.18	0.14
Original slow	1.31	0.17	0.19	0.25
Backward fast	0.99	0.23	0.18	0.13
Backward slow	1.24	0.22	0.17	0.20
**2.0/6.8 S**				
Original fast	3.88	0.72	0.16	0.16
Original slow	4.92	0.71	0.19	0.15
Backward fast	3.96	0.67	0.14	0.07
Backward slow	4.3	0.75	0.16	0.13

An ANCOVA was conducted on the BP with 2 within-subject factors (tempo, backward version) and 1 between-subjects factor (duration), with the arousal and the valence scores for each type of musical pieces as-covariates. This ANCOVA showed a main effect of duration, *F*_(1, 25)_ = 362.72, *p* < 0.05, η ^2^ = 0.94, indicating that the BP was higher for the long than for the short anchor durations. No other factor significantly interacted with duration. More interestingly, there was a significant main effect of tempo, *F*_(1, 25)_ = 8.37, *p* < 0.05, η^2^ = 0.25. This main effect of tempo demonstrates that the BP was lower for the fast than for the slow tempo and therefore indicates that the music was judged longer when played at a faster tempo.

The main effect of backward version was not significant, *F*_(1, 25)_ = 0.72, *p* > 0.05, and the backward version did not interact with any co-variables (all *ps* > 0.05). There was nevertheless a significant tempo × backward interaction, *F*_(1, 33)_ = 5.63, *p* < 0.05, η^2^ = 0.18. This revealed that the music with a fast tempo was judged longer than that with a slow tempo for both the original version, *F*^1^_(1, 35)_ = 60.01, *p* < 0.05, η^2^ = 0.63, and the backward version, *F*_(1, 39)_ = 10.34, *p* < 0.05, η^2^ = 0.21. However, the difference in the lengthening effect between the fast and the slow tempo appeared to be larger for the original than for the backward version, *F*_(1, 34)_ = 13.59, *p* < 0.05, η^2^ = 0.29. In line with results that have been obtained for the assessment of the arousal and valence level of musical pieces, there was a significant interaction between the tempo and the arousal measures for the fast backward music, *F*_(2, 25)_ = 4.39, *p* < 0.05, η^2^ = 0.15, demonstrating that the tempo effect on the BP increased with the arousal scores: The higher the arousal scores, the longer the musical pieces were judged to be. There were also a significant interaction between the tempo and the valence measures, both for the fast and the slow backward version of the musical pieces, revealing that the difference in the lengthening effect between the slow and the fast tempo tended to decrease for the backward version as the pleasantness of the music increased. No other main effect or interaction involving the co-variables was found.

The overall ANOVA run on the WR with tempo, backward version and duration as factors did not reveal any significant effect (all *p* > 0.05). Therefore, the perception of the music distorted time without altering the fundamental ability to discriminate different durations.

Experiment 1 showed a main effect of tempo on time judgment revealing that the musical pieces with a fast tempo were judged longer than those with a slower tempo. There was nevertheless an interactive effect of the version (normal vs. backward version) and tempo of musical stimuli on time judgment. This interaction indicated that the backward version of the music, that was rated as affecting the valence (pleasantness) of the musical pieces, modulated rather than reversed the effect of tempo on the timing of music. Indeed, whatever the stimulus duration ranges (< 2 s >), the musical pieces were always judged longer at the fast than at the slow tempo. However, the magnitude of this lengthening effect due to tempo was larger for the original than for the backward version of musical pieces. In other words, the original or backward version affecting the valence of musical pieces increased or decreased the difference in time judgment between the fast and the slow tempo, without eliminating or reversing the tempo effect.

Our Experiment 1 therefore demonstrates that musical tempo was the major factor affecting time judgments. A musical piece with a fast tempo was systematically judged longer than a musical piece with a slower tempo. Our study with musical pieces thus replicated those of studies using simple click trains, which have showed that a faster click rate produces longer time estimates (e.g., Treisman et al., 1990, 1992). In addition, our results on the emotional evaluation of musical stimuli revealed that the fast pieces of music were systematically judged to be more arousing that the slower pieces. There was also a significant interaction between the tempo and the subjective arousal measures which indicated that the lengthening effect obtained with the fast tempo was, when compared to the slow tempo, related to the increase in the subjective arousal level of the musical pieces. Consequently, the increase in subjective arousal level associated with the fast tempo would be the source of the temporal lengthening effect observed in our study. Such a conclusion would be consistent with the results of numerous studies showing that high-arousing emotional stimuli (facial expressions, images, movies) produce a temporal lengthening effect whereas low-arousing emotional stimuli do not (e.g., Droit-Volet and Gil, 2009; Droit-Volet et al., 2010b, 2011; Gil and Droit-Volet, 2011; Tipples, 2008, 2011). However, the issue of whether the effect of tempo associated with arousal is due to tempo *per se* or to the arousing qualities of the music. We therefore decided to run a second experiment similar to Experiment 1 but with a parameter other than tempo that is also thought to increase the subjective arousal level assessed by the SAM scale (Lang et al., 1999). More precisely, we manipulated the timbre of the musical pieces by playing them in a piano and an orchestral form. Previous studies have manipulated the timbre of musical sounds and demonstrated that the more complex the timbre, the greater the arousal (e.g., Behrens and Green, 1993; Balkwill and Thompson, 1999). Accordingly, piano versions were expected to induce lower arousal than orchestral versions of the same musical pieces. Our hypothesis was that, irrespective of whether arousal level *per se* is the cause of the temporal lengthening, we should observe a temporal lengthening effect for the orchestrated variants similar to those produced by variations in tempo.

## Experiment 2

### Method

#### Participants

The sample consisted of forty new undergraduate students (24 women and 16 men, *mean age* = 21.3; *SD* = 1.54).

#### Material and procedure

The material was similar to that used in Experiment 1 with the exception of the musical stimuli to be timed. To manipulate the arousal induced by the musical stimuli, we changed their instrumentation. In the piano version, only the piano timbre was used. In the orchestral version, additional tracks performed by double bass, woodwind, brass and percussion were included. Increasing the number of virtual performers rendered the music livelier and thus more dynamic. The valence was manipulated in the same way as in Experiment 1 by playing the sound file either normally or backwards. The 5 musical pieces were consequently played either by piano only or with orchestral instrumentation and were run either normally or backwards.

The procedure was also identical to that used in Experiment 1, with a white noise being used for the standard durations presented in the training phase and the musical pieces for the comparison durations presented in the test phase. The test phase consisted of 280 trials presented in 2 blocks of 140 trials each: 10 musical stimulus trials (2 × 5 different musical pieces) for two types of instrumentation (piano vs. orchestral instrumental) and two types of valence (normal vs. backward) for each of the 7 comparison durations. As in Experiment 1, after the bisection task, the participants were again asked to evaluate the emotional qualities of the musical stimuli presented for 1.1 and 4.4 s (mid-point between *S* and *L*) on an affective valence scale ranging from “unpleasant” to “pleasant” and an arousal scale from “calm” to “exciting” (Lang et al., 1999).

## Results and discussion

### Emotional evaluation of musical stimuli

Table 3 shows the results of emotional ratings of the orchestral pieces and corresponding piano versions, presented either forward (normal) or backward. The results of the ANOVA on the pleasantness ratings showed a significant main effect of backward version, *F*_(1, 36)_ = 315.07, *p* < 0.05, η ^2^ = 0.90, thus confirming that the normal music was judged pleasant (7.43) and its backward version unpleasant (2.91). In addition, there was a significant backward × duration interaction, *F*_(1, 36)_ = 15.25, *p* < 0.05, η^2^ = 0.30. This interaction revealed that the difference in affective assessment between a normal piece and its backward version was greater when the presentation duration of the music was long (4.4 s) than when it was short (1.1 s) (5.50 vs. 3.52, *F*_(1, 36)_ = 15.25, *p* < 0.05, η^2^ = 30). The ANOVA also showed that the main effect of orchestration did not reach significance on the pleasantness ratings, *F*_(1, 33)_ = 3.28, *p* > 0.05. This suggests that instrumentation *per se* was not sufficient to modify the pleasant nature of the music. However, there was a significant backward × orchestration × duration interaction, *F*_(1, 36)_ = 5.62, *p* < 0.05, η^2^ = 0.14. For both the short and the long presentation durations, the backward version of the music was systematically judged to be less pleasant whatever its instrumentation (piano or orchestra) (all *p* < 0.05). The only difference in the pleasantness ratings between the piano and the orchestral music was found for the long presentation duration, with the backward version being judged more unpleasant with the orchestral than with the piano sound (2.14 vs. 2.79, *F*_(1, 18)_ = 4.86, *p* < 0.05, η^2^ = 0.21).

**Table 3:** **Mean ratings and standard deviation of arousal and pleasantness (9-point scale) of musical excerpts in original × backward and orchestral × piano conditions for a 1.1 and a 4.4-s duration**.

**Music**	**Arousal**				**Pleasantness**			
	**1.1 s**		**4.4 s**		**1.1 s**		**4.4 s**	
	***M***	***SD***	***M***	***SD***	***M***	***SD***	***M***	***SD***
Original orquestral	6.78	1.28	6.98	1.45	6,56	1.36	7.86	1.32
Original piano	4.29	1.26	5.05	1.84	7.21	1.11	8.07	1.25
Backward orquestral	6.98	1.17	7.04	1.29	3.42	1.6	2.14	1.31
Backward piano	4.45	1.91	4.83	1.80	3.31	1.31	2.79	1.40

In accordance with our hypothesis, the ANOVA on the arousal ratings showed that the orchestral music was judged more arousing that the piano music, 6.95 vs. 4.66, *F*_(1, 37)_ = 139.49, *p* < 0.05, η^2^ = 0.79. In addition, the backward version of the music had no significant effect on subjective arousal, *F*_(1, 37)_ = 0.02, *p* > 0.05. There was no other significant effect. To summarize, by varying the version and instrumentation, we achieved an all but perfect orthogonal manipulation of the valence and the arousing qualities of the musical stimuli. Manipulating the orchestration did indeed selectively affect the arousing values of emotion, while not producing any change in valence.

### Temporal bisection

**Figure 2:**
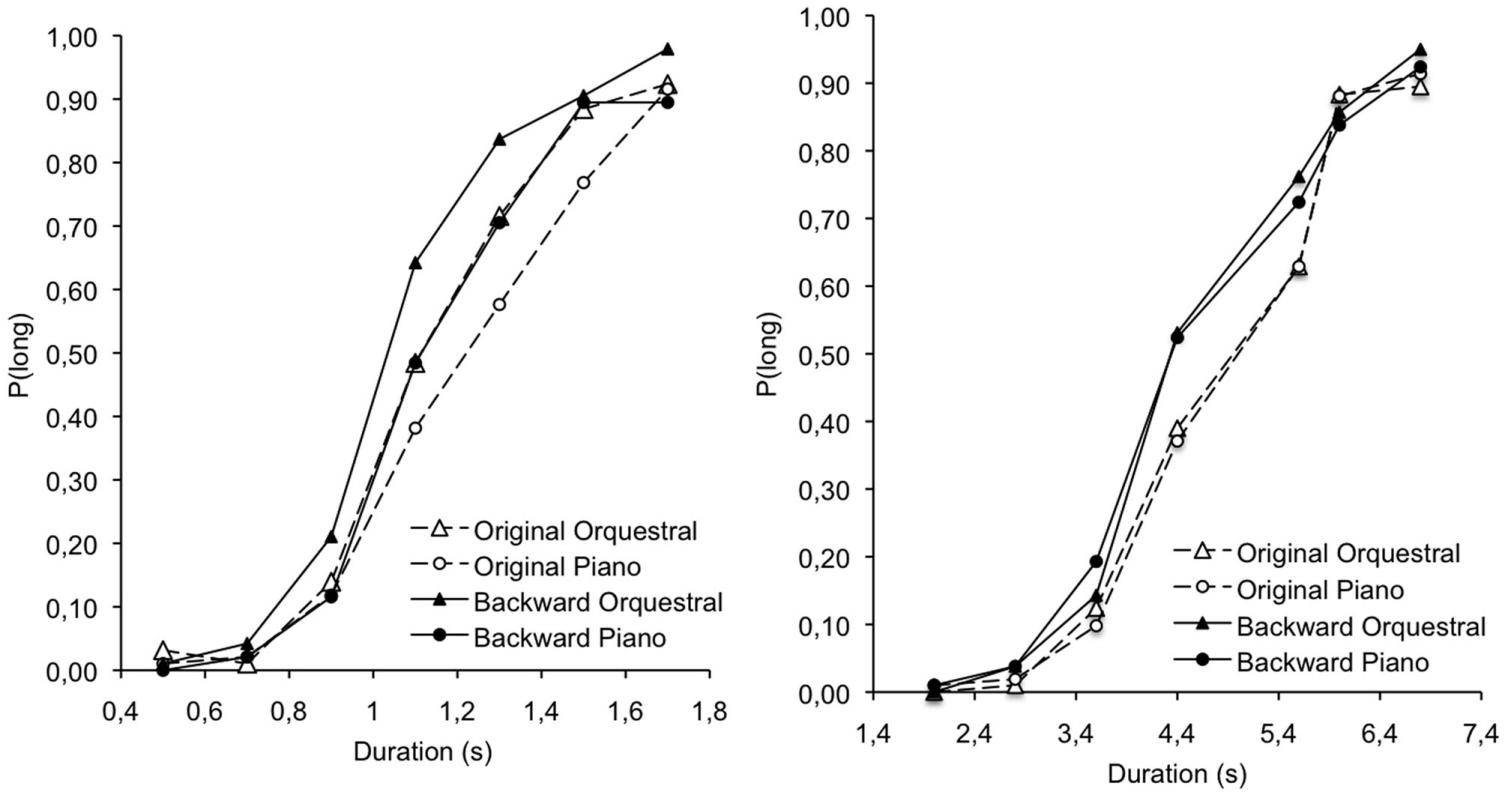
**Proportion of long responses plotted against stimulus duration for the original and the backward version of orchestral and piano music in the 0.5–1.7 and the 2.0–6.8 s duration conditions**.

Figure 2 presents the psychophysical function when the orchestral and piano pieces were played forward and backward in the short and the longer duration range. In contrast to Experiment 1 in which tempo was the major factor modifying time judgment, Figure 2 suggests that the orchestration, although it was also associated with a higher subjective level of arousal, did not affect time judgment. This is confirmed by the results of the ANCOVA performed on the BP (Table 4) with the same factor design as that used in Experiment 1.

**Table 4:** **Means and standard deviation of the Bisection Points and Weber Ratios for original × backward and orchestral × piano music in the 0.5/1.7 and the 2.0/6.8 s duration condition**.

**Music**	**Bisection point**		**Weber ratio**	
	***M***	***SD***	***M***	***SD***
**0.5/1.7 S**				
Original orchestral	1.12	0.15	0.15	0.10
Original piano	1.19	0.18	0.18	0.13
Backward orchestral	1.03	0.09	0.12	0.07
Backward piano	1.13	0.17	0.12	0.08
**2.0/6.8 S**				
Original orchestral	4.54	0.58	0.15	0.10
Original piano	4.62	0.55	0.19	0.18
Backward orchestral	4.34	0.48	0.14	0.09
Backward piano	4.38	0.58	0.15	0.09

As in Experiment 1, the ANCOVA run on the BP revealed a significant main effect of duration, *F*_(1, 27)_ = 595.32, *p* < 0.05, η^2^ = 0.96, with no significant interaction involving this factor. Consequently, the BP was higher in the long than in the short duration range. However, and more interestingly, there are neither main effect of orchestration, *F*_(1, 27)_ = 1.72, *p* > 0.05, nor main effect of backward version, *F*_(1, 27)_ = 0.18, *p* < 0.05. Furthermore, the arousal measures entered into the ANCOVA as covariates were not significant (all *p* < 0.05). The only significant effect was the interaction between the backward version and the valence measures for the original version of the orchestral music, *F*_(1, 27)_ = 6.42, *p* < 0.05, η^2^ = 19. This revealed that the BP increased with the positive valence of the music. In other words, more pleasant the music was judged to be, the shorter the estimate of its duration.

The ANCOVA on the WR failed to reveal any significant effect, except for a significant interaction between the backward version, the orchestration and the arousal measures for the original version of the orchestral music, *F*_(1, 27)_ = 4.67, *p* < 0.05, η^2^ = 0.15. This interaction was due solely to the WR value for the original piano music which increased significantly with the subjective valence level [*r*_(39)_ = 0.36, *p* < 0.05]. In other words, sensitivity to time decreased as the pleasure expressed by the participants when they heard the piano music increased.

To summarize, although the orchestral music was rated as being more arousing than the piano music, our results did not reveal any difference in time perception induced by the musical timbre. Therefore, as we discuss below, both the variations in orchestration and in tempo modified the subjective level of arousal, but only the tempo significantly modified the judgment of time. Finally, when different orchestral pieces were used, only the backward version of the music that modified the affective valence of the music affected time judgments, with the duration of the musical pieces been judged shorter when their positive valence (pleasantness) increased.

In sum, the backward version of musical pieces (original vs. backward) used in our studies to change the emotional valence of the music appeared to produce a shortening effect which, in the case of Experiment 1, modulated the tempo effect on time judgment. However, playing music backwards significantly alters the structure of the music, such as its emotional effect on the perception of time (i.e., temporal shortening) is perhaps specific to this manipulation of the musical pieces. Therefore, to further examine the effect of valence in the temporal judgment of music, we decided to run a third experiment involving the manipulation of other musical parameters that it was considered to modify the emotional valence of music. In a recent study conducted using a similar temporal bisection task as that used in Experiments 1 and 2, Droit-Volet et al. (2010a) tested the emotional valence of musical pieces by presenting the same pieces in two variants: a major key for positive valence and a minor key for negative valence. However, as we explained in our Introduction, they did not report any significant effect of mode on the perception of time with different duration ranges. In Experiments 1 and 2, we manipulated the valence of the music by inverting the amplitude envelope of the musical pieces (forward vs. backward version). Another approach consists in contrasting tonal and atonal music. Using a retrospective temporal judgment paradigm, Kellaris and Kent (1992) made a pop song played in the major or minor mode and lasting 2.5 min atonal by changing the pitch of appropriates tones. The participants judged the piece played in the major mode (associated with happiness) as lasting longer (3.45 min) than that played in the minor mode (3.07 min) or in an atonal variant (2.95 music). The authors therefore concluded that the strongest valence effects were found when major and atonal versions of the same music were contrasted. Consequently, in Experiment 3, we used a temporal bisection task to examine the differences in time perception caused by tonal and atonal pieces of music.

## Experiment 3

### Method

#### Participants

Forty new undergraduate students (22 women and 18 men, *mean age* = 24.2, *SD* = 2.03) participated in this experiment.

#### Material and procedure

The same 5 musical pieces as in Experiment 1 were used, but now in their tonal and atonal versions. The tonal and atonal versions of each piece had identical musical parameters such as rhythm, meter, and melodic contour. All the stimuli (tonal and atonal) were played at a fast tempo of 108 beats per min. They differed only in the fact that the atonal version contained pitches that did not belong to a unique key, thus creating dissonant intervals.

The procedure was again identical to that employed in the previous experiments, with a white noise being used to indicate the standard durations presented in the training phase and the pieces of music being used for the comparison durations presented in the test phase. However, in the test phase, only two types of music were used (atonal vs. tonal). The test phase thus consisted of 140 trials subdivided into 2 blocks of 70 trials each: 10 trials (5 musical pieces × 2) in their tonal and atonal versions for each of the 7 stimulus durations. After the bisection task, the participants were again asked to evaluate the emotional qualities of the stimuli on both an affective valence and an arousal scale.

## Results and discussion

### Emotional evaluation of musical stimuli

Table 5 displays the average emotional ratings provided by the participants. Not surprisingly, tonal music was considered more pleasant than atonal music irrespective of stimulus duration The analysis of variance (ANOVA) run on the pleasantness ratings showed a significant main effect of tonality, *F*_(1, 28)_ = 156.57, *p* < 0.05, η^2^ = 0.85, and no significant effect of duration, *F*_(1, 28)_ = 1.55, *p* > 0.05, or significant duration × tonality interaction, *F*_(1, 28)_ = 1.44, *p* > 0.05. By contrast, the ANOVA on the arousal ratings did not reveal any significant effect: Tonality, *F*_(1, 29)_ = 0.01, Duration, *F*_(1, 28)_ = 3.34, Tonality × Duration, *F*_(1, 29)_ = 3.24, all *p* > 0.05. This finding suggests that the change in pitch structure primarily affected only the valence of the pieces, with atonal music being judged more unpleasant than tonal music.

**Table 5:** **Mean and standard deviation of ratings of arousal and pleasantness (on a 9-point scale) for musical excerpts in tonal and atonal conditions for a 1.1 and a 4.4-s duration**.

**Music**	**Arousal**				**Pleasantness**			
	**1.1 s**		**4.4 s**		**1.1 s**		**4.4 s**	
	***M***	***SD***	***M***	***SD***	***M***	***SD***	***M***	***SD***
Tonal	4.92	1.62	6.4	1.71	7.2	1.11	8.1	0.67
Atonal	5.84	1.39	5.38	1.72	3.01	1.42	3.02	1.98

### Temporal bisection

**Figure 3:**
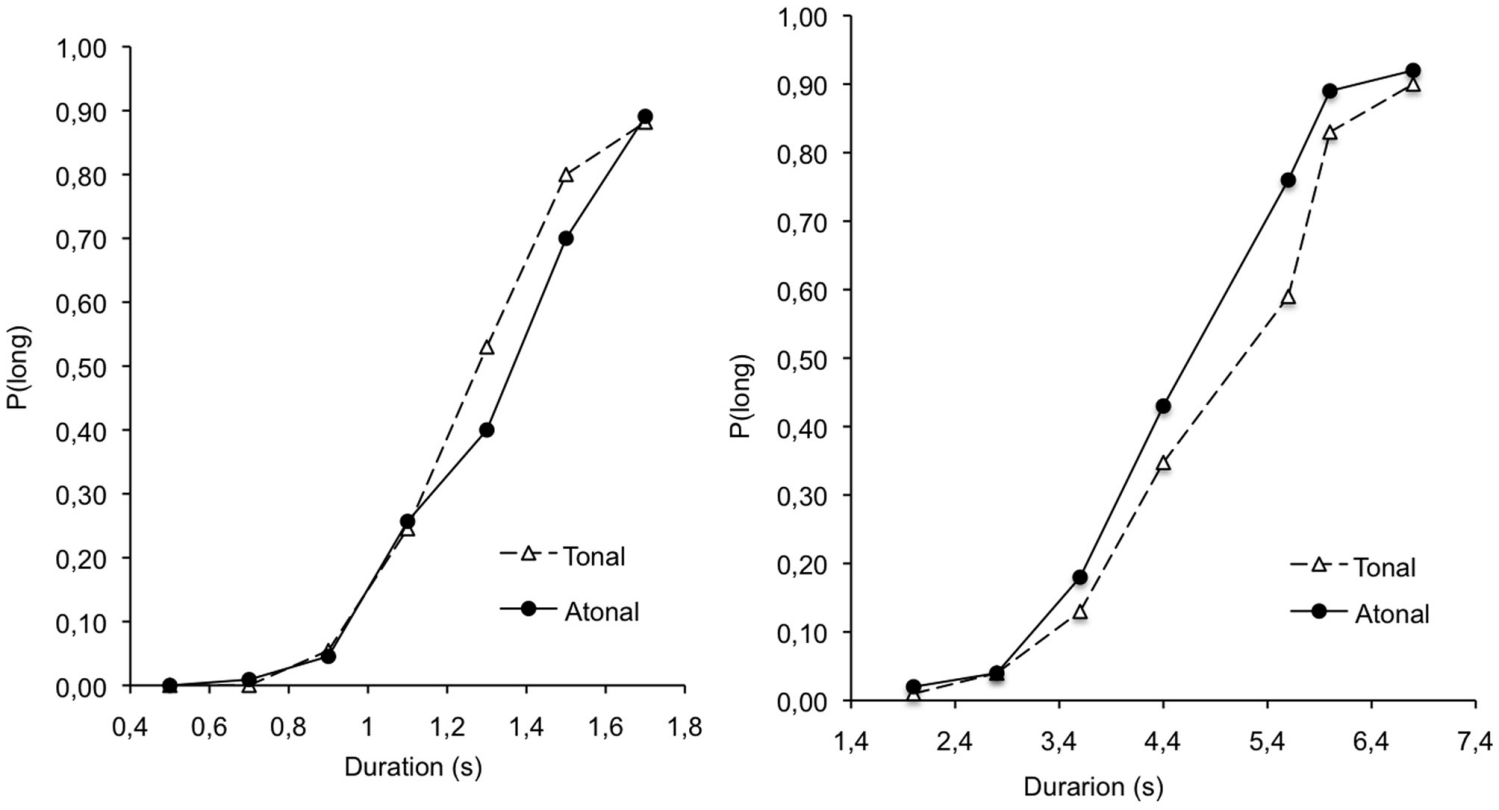
**Proportion of long responses plotted against stimulus duration for the tonal and atonal music in the 0.5–1.7 and the 2.0–6.8 s duration conditions**.

Figure 3 indicates the psychophysical functions for the two types of music. This Figure suggests that, in line with the results found in Experiment 1, there was a tonality effect for the long duration range (2.0/6.8-s), with the tonal pleasant music being perceived as lasting for less time than the atonal pleasant music. However, no clear-cut effect of this type seems to be observed for the very short duration range (0.5/1.7-s).

Table 6 presents the BP and WR calculated using the regression method as in Experiment 1. The ANCOVA was performed on the BP and the WR with duration as between-subjects factor, music as within-subjects factor, and arousal and valence scores as co-variables. The ANCOVA on the BP showed a significant main effect of duration, *F*_(1, 24)_ = 585.96, *p* < 0.05, η^2^ = 0.96, as in the previous experiments. There was also a significant main effect of tonality, *F*_(1, 24)_ = 4.84, *p* < 0.05, η^2^ = 0.17, as well as a significant tonality × valence interaction, *F*_(1, 24)_ = 5.38, *p* < 0.05, η^2^ = 0.18. The BP was thus significantly higher for the tonal music than for the atonal music, indicating that the duration of the tonal music was judged shorter than that of the atonal music. In addition, this shortening effect increased with emotional valence, i.e., as the assessment of the music as pleasant increased.

**Table 6:** **Mean and standard deviation of the Bisection Points and Weber Ratios for tonal and atonal music in the 0.5/1.7 and the 2.0/6.8 s duration condition**.

	**Bisection point**		**Weber ratio**	
	***M***	***SD***	***M***	***SD***
**0.5/1.7 S**				
Tonal	1.25	199	0.09	0.03
Atonal	1.31	192	0.09	0.04
**2.0/6.8 S**				
Tonal	4.73	796	0.13	0.07
Atonal	4.25	615	0.11	0.09

The ANCOVA on the WR also found a main effect of emotion valence for the tonal music, *F*_(1, 21)_ = 4.85, *p* < 0.05, η^2^ = 0.19, indicating that sensitivity to time decreased with the increase in the positive valence of the music. The ANCOVA did not show any other significant effect (tonality, *F*_(1, 24)_ = 0.03, tonality × duration, *F*_(1, 24)_ = 0.10, duration, *F*_(1, 39)_ = 0.004, all *p* > 0.05). This lack of significant effect for the WR involving duration in Experiment 3 as well in Experiments 1 and 2 confirmed that Weber's law holds for the temporal judgment of music as well as for that of other stimuli (Wearden and Lejeune, 2008). In conclusion, the manipulation of physical properties of musical pieces produced time distortions without impairing the fundamental ability to discriminate different durations.

In sum, the results of Experiment 3 revealed that the stimulus durations were judged shorter with the tonal than with the atonal music. As the tonality affected the emotional valence with the tonal music being judged more pleasant than the atonal music, our results demonstrated that hearing a pleasant music produced a temporal shortening effect compared to an unpleasant music. Consequently, modulating the emotional valence of music by changing its tonality or by inversing its amplitude envelope (backward version) produced a similar temporal shortening effect for different duration ranges.

## General discussion

Numerous studies have addressed the influence of emotion on the perception of time (for reviews, see Droit-Volet and Meck, 2007; Droit-Volet, 2013; Droit-Volet et al., 2013). However, most of these have used emotional visual stimuli (i.e., emotional facial expressions, pictures from IAPS). Only two experiments, conducted by Noulhiane et al. (2007) and Mella et al. (2011), has been undertaken with sounds from the International Affective Digital Sounds (IADS, Bradley and Lang, 1999). The results of these 2 experiments showed that the emotional sounds were judged longer than the neutral sounds, and more so in the case of the negative compared to the positive sounds. These results were explained within the theoretical framework of the internal clock models (Treisman, 1963; Gibbon, 1977; Gibbon et al., 1984) in terms of arousal effects which speed up the internal clock rate. According to the internal clock models, when the speed of the internal clock increases, more temporal units (pulses) are accumulated and time is judged longer. As in most studies of time and emotion, Noulhiane et al. (2007) therefore concluded that “physiological activation is the predominant aspect of the influence of emotions on time perception, as all emotional stimuli regardless of their self-assessed valence are perceived as being longer than neutral ones” (p. 702).

However, emotional sounds differ from other emotional stimuli (visual) because they are dynamic stimuli involving different parameters that evolve through time. Without specific experimental manipulations of these different parameters, it is thus difficult to identify the real sources of temporal distortions in response to these sounds. For instance, musical pieces played in a major key at a fast tempo are judged happier than those played in a minor key at a slow tempo (e.g., Peretz et al., 1998; Fritz et al., 2009). More specifically, in the case of the perception of time, the tempo in itself must affect the speed of the internal clock independently of emotional effects. Many different studies have shown that a simple sequence of periodic stimuli (clicks, flickers) increases temporal estimates (for a review, see Wearden et al., 2009). Wearden et al. (2009) concluded that the click train effect on the perception of time due to a speeding up of the internal clock is one of the most robust effects to be observed in time psychology. However, the use of music provides an elegant way of manipulating two dimensions while keeping a number of other parameters constant. The present study addressed this issue by manipulating, in Experiments 1 and 2, two different dimensions of arousal (tempo and timbre) as well as a parameter associated with emotional valence (backward vs. forward music). Our results revealed that variations in tempo are indeed associated with different subjective levels of arousal, with music played at a faster tempo being judged as more arousing that played at a slow tempo. In the same way, orchestration was found to affect arousal level, with orchestral music being judged to be more arousing than piano music when the tempo of these two types of music was held constant. Nevertheless, in our temporal bisection studies we found that, although these two musical parameters affected the subjective level of arousal, only the tempo significantly modified the perception of time. Indeed, in Experiment 1, the psychophysical functions were systematically shifted toward the left, with the BP being lower for the fast than for the slow music, thus indicating that the fast music was judged as lasting longer than the slow music. By contrast, in Experiment 2, no significant effect of timbre on the perception of time was observed although the orchestral music was judged to be more arousing than the piano music. In conclusion, as far as music is concerned, tempo is one of the major factors associated with the emotional arousal that leads to distortions in temporal judgments. In other words, the physical properties of music plays a fundamental role in the time distortions associated with emotion.

In addition, Noulhiane et al. (2007) have suggested that, compared to physiological activation, the valence of emotional sounds has only a small influence on the perception of time. This idea finds support in the fact that a temporal lengthening effect, related to the physiological activation resulting from accelerated tempo, was systematically observed in our study whatever the emotional valence of the musical pieces and irrespective of their duration (shorter or longer than 2 s). However, the results of our study also revealed an effect of emotional valence on judgments of the duration of musical pieces, even when stimulus durations were particularly short. Indeed, regardless of the type of musical property that changed the emotional valence (the backward version, the tonality), our studies demonstrated that listening to music with a positive valence led to shorter time estimates. This finding is entirely consistent with the results of previous studies in which participants were asked to evaluate the duration of a long period of music (e.g., Yalch and Spangenberg, 1990; Kellaris and Kent, 1994). Finally, emotional valence rated in terms of pleasure (unpleasant vs. pleasant) seems to be a more sensitive index of emotional effects on time judgments than emotional valence rated in terms of mode (sad vs. happy music) (Bueno and Ramos, 2007; Droit-Volet et al., 2010a,b). As argued by Droit-Volet et al., 2010a, sad music can be also judged as pleasant.

The question that must now be asked is: Why did the emotional valence of the music produce a shortening effect on time judgments, whereas arousal produced a contrasting lengthening effect? As explained above, the lengthening effect obtained with arousal/tempo is probably due to an automatic speeding up of the internal clock. In contrast, the effect of valence (unpleasant vs. pleasant) might call on controlled attentional processes which are linked to the awareness of pleasure experienced when listening to pleasant music. According to attentional models of timing, the temporal and the non-temporal processors compete for the same pool of attentional resources (Thomas and Weaver, 1975; Zakay, 1989; Zakay and Block, 1996, 1998). Temporal units (pulses) that underpin the representation of time would be lost when attentional resources are distracted away from the processing of time, thus resulting in a shortening effect. This assumption, made by the attention-based models of timing, has been widely validated by the results of numerous studies that have used the dual-task paradigm (e.g., Fortin and Breton, 1995; Casini and Macar, 1997; Gautier and Droit-Volet, 2002; Coull et al., 2004). The results of our study, which showed that hearing musical pieces of positive valence shortened the passage of time, are thus consistent with this attentional assumption. Consequently, hearing pleasant music seems to divert attention away from time processing. In other words, time flies when subjects listen to pleasant music. In addition, our results in Experiment 1 revealed that this attention-related shortening effect was greater in the case of low-arousing music with a slow tempo. However, further experiments must be run to gain a better understanding of the effect of the interaction between the two emotional dimensions of the music (valence and arousal) on the timing of music.

In conclusion, the originality of our study lies in the fact that it reveals that the arousal and valence-related properties of a musical stimulus have an interactive effect on time perception. However, our study also showed that the critical factor responsible for producing time distortions was the tempo of the music. In consequence, the emotional effect of music on the perception of time is intrinsically linked to the temporal dynamic of music, i.e., its musical tempo. It is therefore particularly important to continue our investigation of music in order to better understand the way emotions affect time perception because emotional music has dynamic temporal properties which are not present in visual emotional stimuli.

### Conflict of interest statement

The authors declare that the research was conducted in the absence of any commercial or financial relationships that could be construed as a potential conflict of interest.

## Footnotes

^1^Bonferroni corrections were applied for all comparisons.
